# Human Artificial Chromosomes and Their Transfer to Target Cells

**DOI:** 10.32607/actanaturae.11670

**Published:** 2022

**Authors:** S. V. Ponomartsev, S. A. Sinenko, A. N. Tomilin

**Affiliations:** Institute of Cytology Russian Academy of Sciences, St. Petersburg, 194064 Russia; Institute of Translational Biomedicine, St. Petersburg State University, St. Petersburg, 199034 Russia

**Keywords:** human artificial chromosomes, microcell-mediated chromosome transfer, tetracycline operator, transformation-associated recombination

## Abstract

Human artificial chromosomes (HACs) have been developed as genetic vectors with the capacity to carry large transgenic constructs or entire gene loci. HACs represent either truncated native chromosomes or de novo synthesized genetic constructs. The important features of HACs are their ultra-high capacity and ability to self-maintain as independent genetic elements, without integrating into host chromosomes. In this review, we discuss the development and construction methods, structural and functional features, as well as the areas of application of the main HAC types. Also, we address one of the most technically challenging and time-consuming steps in this technology – the transfer of HACs from donor to recipient cells.

## INTRODUCTION

Human artificial chromosomes (HACs) were conceived primarily as expression vector systems for the transfer of transgenes into eukaryotic cells. To date, many vector systems have been created that differ in their main characteristics: (1) the ability to integrate into the chromosomes of host cells or remain in episomal form; (2) the genetic capacity that restricts the maximum transgene size; (3) and the method of vector delivery. Integrating vectors are inserted into the host cell’s DNA and, consequently, are inherited by daughter cells. The disadvantages of these vector systems include their random integration into the genome, which comes with the risk of insertional mutagenesis and epigenetic repression of transgene expression. Integrating vectors include linearized plasmids and vector systems based on retroviruses [1 , 2, 3] and transposons, such as piggy-Bac, Sleeping Beaty, and Tol2 [4, 5, 6].

Non-integrating vectors are present in episomal state in the host cells. During cell division, these vectors are unevenly distributed between daughter cells and gradually lost. These systems are convenient for transient transfection of cells, but they are not suitable for long-term expression of transgenes. These vector systems are exemplified by circular plasmids and vectors based on adenoviruses, alphaviruses, herpesviruses, baculoviruses, poxviruses, and bacteriophages [1, 2, 7]. An important parameter of vector systems is their capacity that is defined as the maximum size of an inserted transgene. Plasmids can be used to transfer transgenes of up to 20 kilo base pairs (kbp) in length. Transposon-based vectors can be used to deliver transgenic DNA of up to 9 kbp, whereas viral DNA-based vector systems can accommodate transgenes of up to 150 kbp [12]. There are various methods for the transfer of expression vector systems into target cells. Plasmids and DNA transposon-based vectors are transferred using calcium phosphate transfection, electroporation, lipofection, sonoporation, microinjection, magnetofection, and the so-called gene gun. Delivery of viral DNA-based vectors, which is called transduction, is performed using the host cell infection mechanisms typical of viruses.

Human artificial chromosomes are vector constructs that possess the following crucial chromosomal characteristics: (1) the ability to self-maintain autonomously, i.e., as an additional chromosome, in the cell and (2) the ability to replicate and be transmitted to both daughter cells during cell division. Thus, the use of HACs skirts the risks of insertional mutagenesis and ensures a stable expression of transgenes. A unique feature of HACs is their ultra-high capacity that enables the transfer of transgenes up to several-million-base-pairs long, in particular entire gene loci with cis-regulatory elements, which ensures an accurate expression of endogenous loci. Although many structurally diverse HACs have been developed to date, these vector systems are still being intensively improved and modified [8, 9, 10, 11, 12, 13]. Two approaches are used to produce HACs. The first is the so-called top-down approach that is based on the production of HACs from native chromosomes by their maximal truncation, leaving only the centromeric and telomeric regions that are necessary for their stable replication in the cell [14 , 15, 16]. The second is the synthetic bottom-up approach that is used to produce linear or circular HACs through the synthesis and assembly of large regions of pericentromeric alpha-satellite DNA in vitro [13, 17, 18, 19]. It should be noted that, despite the obvious advantages of HACs over other vector systems, there is a number of technical limitations standing in the way of their extensive use both in scientific research and in biomedical applications. One of the main limitations of the system is the inefficiency and laboriousness of the methods used to transfer HAC into target cells. This review describes different HAC types, methods of delivery into cells, and prospects for the application of these ultra-high capacity episomal vectors in medical practice.

## MAIN HAC TYPES AND METHODS FOR THEIR PRODUCTION

**HACs produced by reduction of native human chromosomes**

**Fig. 1:**
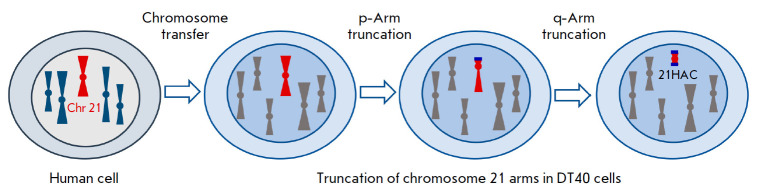
Schematic representation of 21HAC assembly using telomere-associated chromosome truncation. Human chromosome 21 was transferred to DT40 cells. Then, telomeric sequences (shown in blue) were inserted into the pericentromeric site using homologous recombination, which led to truncation of the chromosome. Thus, the 21HAC was generated using successive truncation of chromosome arms and their replacement with telomeric sequences

Eukaryotic chromosomes can be truncated using telomere-associated chromosome fragmentation (TACF) [20]. To date, these are the most characterized and improved HACs in terms of their use as stable expression-vector systems. The top-down approach enables truncation of chromosome arms and their replacement with new telomere-containing regions that are inserted into selected loci using homologous recombination. The resulting HACs may contain some cryptic genes and non-coding sequences, but they always involve the elements necessary for their stable maintenance in the cell nucleus (telomeres) and equal distribution between daughter cells during cell division (centromeres). For site-specific integration of transgenes into these constructs, appropriate sequences, e.g., loxP sites, which mediate transgene integration through Cre-dependent recombination, are preliminarily introduced in the transgenes. Also, HACs often contain selective markers that enable positive selection of HAC-containing cells. The use of TACF has enabled the production of artificial chromosomes based on human chromosomes 14 [21] and 21 [16, 22, 23] and mouse chromosome 11 (mouse artificial chromosomes) [24]. The HAC based on human chromosome 21, 21HAC [16], which was produced in several stages (Fig. 1), is the most technically advanced construct to date. Native human chromosome 21 was first transferred into chicken DT40 cells suitable for homologous DNA recombination [25]. Then, the p-arm was deleted from the transferred chromosome using TACF; for that purpose, a telomeric sequence was inserted into the pericentromeric region using homologous recombination. Along with the telomeric sequence, a selective marker was also inserted; the marker enables selection of the cells in which recombination has occurred. The q-arm was deleted in a similar way (Fig. 1). In addition to the telomeric region, the loxP site, a fragment of the hypoxanthine-guanine-phosphoribosyltransferase (HPRT) gene, and other elements were also introduced into the 21HAC. Using sequencing, the resulting 21HAC was shown to contain not only the centromeric region and inserted elements, but also an insignificant amount of residual genetically inert material [26]. The resulting HAC was transferred from DT40 cells to CHO cells for the final stage of HAC assembly, which includes loading of an appropriate transgene using site-specific recombination, as well as maintenance and production of this HAC. Then, the HAC was transferred to the target cells using microcell-mediated chromosome transfer (MMCT) as described below.

There are several 21HAC modifications that have been generated using different selective markers: the green fluorescent protein (GFP) gene, thymidine kinase (tk) gene of the herpes simplex virus, and resistance genes to neomycin, hygromycin, and blasticidin [26]. There is also a 21HAC containing a multi-integrase locus involving the loxP, FRT, *φ*C31attP, R4attP, TP901-1attP, and Bxb1attP sites [23]. This HAC has been used in various research and gene therapy models [8], the development of expression vectors for correction of Duchenne muscular dystrophy [22, 27, 28], hemophilia A [29], and the reprogramming of mouse embryonic fibroblasts [30].

**Satellite-based HACs**

**Fig. 2:**
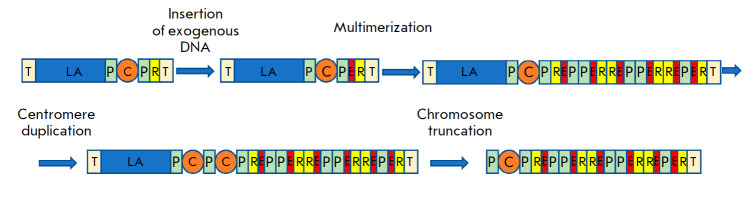
Diagram of generation of a satellite DNA-based artificial chromosome (SATAC). Exogenous DNA bearing site-specific recombination sites, a selective marker, and other sequences is inserted into the pericentromeric region of an acrocentric chromosome using homologous recombination. This insertion results in the amplification of pericentromeric, ribosomal, and exogenous DNA regions. Centromere duplication is followed by chromosome truncation, which leads to HAC formation. Symbols: T – telomere; LA – long arm of the acrocentric chromosome; P – pericentromeric region; C – centromere; R – ribosomal DNA; E – exogenous DNA

Another HAC type generated using the top-down approach is produced by inserting a transgene into the ribosomal DNA gene cluster in the short arms of acrocentric chromosomes [31, 32] (Fig. 2). This insertion may be associated with replication errors, which results in the formation of long inverted repeats [33]. Along with this, the centromere doubles, the chromosome breaks off, and the short arm fragment forms a separate chromosome that behaves as an independent replicative unit [34, 35] and enables a stable expression of the inserted transgene [36]. The resulting HACs, called satellite DNA-based artificial chromosomes (SATACs), are isolated from donor cells using flow cytofluorometry and transferred to target cells using dendrimers and cationic particles [37] or microinjection [38, 39]. Mouse embryonic stem cells (ESCs) with transferred SATACs were able to participate in normal embryonic development [38, 40].

**Alphoid HACs**

A fundamentally different way to create HACs is based on the synthesis of extended nucleotide sequences possessing the main functions of chromosomes. The main difficulty in the bottom-up approach is the design of a functional artificial centromere sequence. In human chromosomes, this sequence is known to consist of alpha-satellite DNA tandem repeats 230 kbp to several mega base pairs (Mbp) in length [41]. These sequences are very difficult to clone, due to spontaneous recombination [42]. The first successful attempt to clone human centromeric DNA was undertaken in 1997 [43]. Using multiple ligation rounds, long (several kbp) alpha-satellite DNA tandem repeats from the centromeres of human chromosomes 17 and Y were cloned into a bacterial artificial chromosome (BAC), resulting in repeats of up to 173 kbp in length. Ligation of these fragments provided human alpha-satellite DNA sequences more than 1 Mbp in length. The cloned centromeric repeats, telomeric sequences, and human genomic DNA fragments were transferred to human fibrosarcoma HT1080 cells, where they nonspecifically recombined with each other. In some cases, small HACs were formed, which remained stable in the cell nucleus and were inherited by both daughter cells. Thus, the fundamental possibility of de novo HAC assembly was shown for the first time, which gave impetus to further research in this direction.

**Alphoid^tetO^-HAC**

**Fig. 3:**
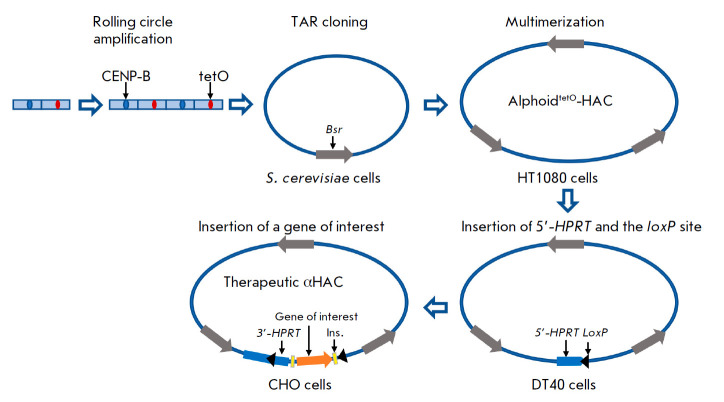
Representation of alphoidtetO-HAC assembly. At the first step, a tandem array comprising two units is synthesized: one unit is a 170-bp *CENP-B*-*box*-containing (blue oval) alphoid repeat from the human chromosome 17 centromere, and the second unit contains the same repeat in which the *CENP-B-box *is replaced with the tetracycline operator (*tetO*, red oval). Rolling circle amplification of the array produces a 10-kbp fragment. These fragments are cloned by TAR cloning in yeast cells, which provides a 50-kbp circular construct containing the blasticidin resistance gene (*Bsr*, gray arrow). The circular construct is multimerized in HT1080 cells, which results in the formation of a 1.1-Mbp alphoidtetO-HAC. Fusion of HT1080 cells with DT40 cells (black arrow) is accompanied by the insertion of the *loxP *site into the HAC. The alphoidtetO-HAC is transferred to CHO cells, where the construct is loaded with a gene of interest (orange arrow), together with the flanking insulator sequences (yellow boxes) and a 3’ fragment of the *HPRT *gene (blue line). The alphoidtetO-HAC is transferred to target cells using MMCT

This HAC type is assembled using alpha-satellite DNA amplification by rolling circle replication and transformation-associated recombination (TAR) [44, 45, 46] (Fig. 3). The former method is used to multimerize a DNA dimer, one monomer of which is a 170-bp alpha-satellite DNA sequence from human chromosome 17, which contains the CENP-B box (required for the assembly of the kinetochore complex), and the other monomer is the same sequence where the CENP-B box is replaced with the tetO site. Next, the multimerized alpha-satellite DNA repeats and linearized vector for TAR cloning were transferred to Saccharomyces cerevisiae yeast cells, where the DNA repeats recombined with each other. This event resulted in the formation of longer sequences that were inserted into the TAR vector containing the blasticidin resistance gene [47, 48, 49, 50]. The resulting constructs were transferred to HT1080 human fibrosarcoma cells, where they additionally multimerized and formed circular DNA molecules 1–2.5 Mbp in length. Thus, the main sequence of these molecules was centromeric alpha-satellite DNA. The produced genetic constructs were shown to be stable and act as independent genetic elements in cells: i.e., they were HACs [51, 52]. For further genetic manipulations, HT1080 cells containing the resulting HACs were fused with DT40 cells that are commonly used for homologous recombination of genetic elements. This resulted in the formation of HACs with an inserted loxP site and a 5’-fragment of the HPRT gene. These HACs were transferred to HPRT-mutant CHO cells using the MMCT procedure (see below). A desired transgene can be inserted into HACs within these cells by Cre-mediated recombination at the loxP site. For this purpose, this transgene containing regulatory sequences, flanking insulators, and a 3’-fragment of the HPRT gene are inserted into a HAC (Fig. 3). Thus, correct transgene insertion into a HAC is accompanied by HPRT gene restoration, which enables selection of target clones in the presence of hypoxanthine-aminopterin-thymidine (HAT). It should be noted that the presence of tetO sites in alphoidtetO HAC enables, if necessary, deletion of these chromosomes during cell division. For this purpose, cells are induced to express TetR repressors that bind tetO, repress centromeric chromatin, and, thus, inhibit kinetochore complex formation [18, 52, 53, 54].

Full-length genes containing their own cis-regulatory sequences were transferred to target cells using alphoidtetO HACs, and stable expression of these genes was demonstrated [19, 54, 55, 56]. In our studies, GFP-expressing alphoidtetO HACs were transferred to mouse ESCs. Teratomas and chimeric mice generated using these cells stably maintained this HAC and expressed GFP in differentiated progeny of ESCs [57]. Also, the alphoidtetO HAC was successfully transferred to human iPSCs that retained pluripotent properties in the presence of this HAC [58]. Thus, we have shown that the introduction of alphoid^tetO^ HACs does not affect the pluripotent properties of mouse and human cells. Finally, we created an alphoid^tetO^ HAC expressing blood coagulation factor VIII, which may be further used to develop stem cell-based gene therapy methods for the treatment of hemophilia A [56].

**Bacterial and yeast artificial chromosome-based HACs**

The first study on the construction of yeast artificial chromosome (YAC)-based HACs was performed in 1998 [59]. A 100-kbp human chromosome 21 centromeric DNA sequence containing multiple CENP-B protein binding sequences (CENP-B boxes) was cloned into YACs. The resulting construct was modified in yeast cells by truncating the distal regions and replacing them with the telomeric regions of human chromosomes. Additionally, selective markers were inserted, after which the constructs were transferred to human fibrosarcoma HT1080 cells using lipofection (Fig. Fig. 44). In these cells, YACs underwent further multimerization, which led to the formation of 5 Mbp HACs that were stable in HT1080 cells and were stably inherited during cell divisions [13].

**Fig. 4:**
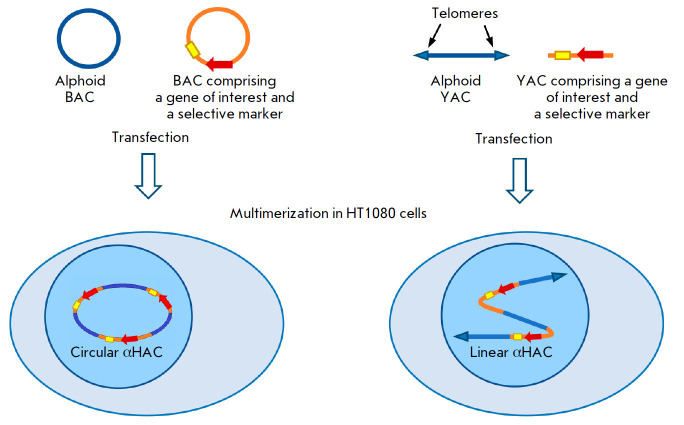
Diagram of HAC assembly using bacterial and yeast artificial chromosomes (BACs and YACs, respectively). Circular BACs or linear YACs are used to assemble two vectors: one vector contains alphoid DNA, and the other contains a gene of interest. These constructs co-transfected into HT1080 cells undergo recombination and multimerization to form circular or linear alphoid HACs (αHACs)

Later, there were successful attempts to generate BAC-based HACs [60] (Fig. 4). In this approach, HT1080 cells were co-transfected with a BAC that contained human chromosome 21 centromeric regions and sequences comprising full-length genes and their regulatory elements. In these cells, there was recombination of the introduced DNA molecules, followed by their subsequent multimerization, which led to the formation of circular HACs that stably replicated, were inherited by daughter cells, and maintained expression of target genes. Circular BAC-based HACs and linear YAC-based HACs were shown to be successfully transferred to mouse ESCs. Chimeric animals were produced by injection of these cells into blastocysts; differentiated progeny of ESCs stably maintained both a HAC and expression of a transgene introduced with the HAC [60].

that carried the elements necessary for its use as an expression vector system, which included a sequence for site-specific recombination, a selective marker, and transcriptional insulators [61]. This HAC was used to develop vector constructs with different sites for site-specific recombination [62]. These types of HACs were used to develop a number of gene therapy models: transfer of a globin gene cluster to K562 cells [63], conducting cell immortalization [13, 64], generating a transgenic mouse model for Down syndrome [65], and identifying a genetic locus that provides silencing of the HLA-G gene in most tissues [66]. Finally, the possibility of transferring this HAC to human iPSCs was confirmed, opening a possibility of their applications in gene therapy [67].

**HSV-1 amplicon-based HACs**

A unique method for a direct HAC assembly in mammalian cells with the use of a herpes simplex virus type 1 (HSV-1) amplicon-based vector has been proposed [68]. This vector contains the Pac signal, which is necessary for its assembly into the viral capsid, and the viral replication origin, OriS [69]. The transgene-containing vector and two additional genetic constructs were co-transfected into green monkey cells to produce the vector amounts necessary for the transfection and package of the vector into the viral capsid. These additional constructs were expression vectors, one of which contained most of the HSV-1 genes required to assemble the viral capsid and pack the viral DNA into it. The other vector contained the ICP27 gene required to regulate the expression of viral genes. Both accessory constructs lacked the Pac and OriS signals, which prevented them from replicating and packaging into the viral capsid. Viral amplicon vectors are able to accommodate a transgene of about 152 kbp in length [70, 71].

**Fig. 5:**
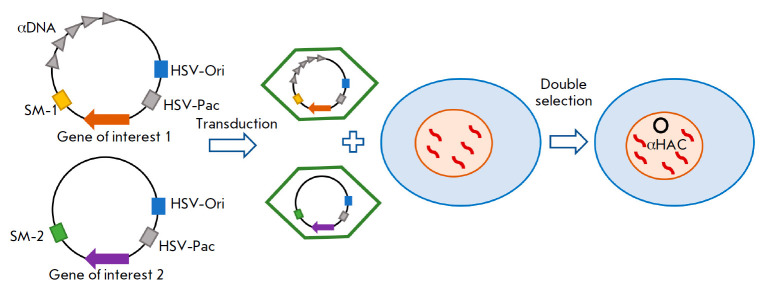
Schematic representation of HSV-1 amplicon-based HAC assembly. Two vectors were constructed on the basis of herpes simplex virus (HSV-1) amplicons. One vector contained the origin of the replication signal (*Ori*), viral capsid packaging signal (*Pac*), selective markers (SM-1 and 2), and genes of interest. The second vector contained a 120-kbp sequence comprising alpha satellite repeats from human chromosome 17 (αDNA). The resulting viruses were co-transduced into target cells. Double selection was used to select cells in which two vector constructs recombined to form the target alphoid HAC (αHAC)

To assemble a HAC, human chromosome 17 and 21 centromeric sequences, a target gene, and selective markers were introduced into a BAC-containing OriS and Pac signals [68]. This vector and two auxiliary plasmids were transferred to green monkey cells, which ensured production of the vector and its packaging into the viral capsid that was then transduced into human cells (Fig. 5). The produced genetic construct was shown to act as a HAC, being maintained during cell division and providing stable transgene expression. Also, mitotic stability of the HAC was found to be mediated by an alpha-satellite sequence in a 40-kbp vector. Given that the maximum capacity of HSV-1-based vectors is 152 kbp, and that the centromeric region length is approximately 42 kbp, a target transgene of up to 110 kbp in length can be inserted into the considered HAC. An important indication that the HSV-1 replicon-based HAC may be used in cell therapy in the future was its successful transfer to human ESCs [72] and iPSCs [73].

Recently, the method for assembling this HAC has been improved. Human cells were transduced with two different vectors, one of which contained the alpha satellite sequence of human chromosome 17, and the other contained target genes [73]. When these vector constructs met in the cell nucleus, they recombined with each other to form a stable HAC with double the size of the initial one (Fig. 5). Therefore, this approach can be used to transfer transgenes of up to 260 kbp in length [11].

**Methods of HAC transfer to target cells**

**Fig. 6:**
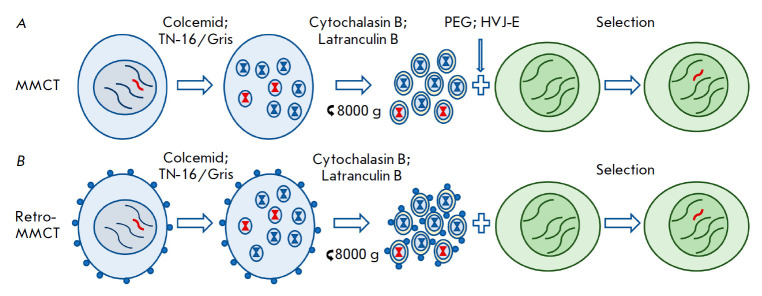
Methods for HAC transfer from donor to recipient cells using MMCT. Donor cells are depicted as blue ovals, and recipient cells are shown as green ovals. HACs are marked in red. (*A*) The original MMCT method. At the first step, cells are treated with colcemid or TN-16/griseofulvin (Gris) to produce metaphase micronuclei. After treatment of cells with cytochalasin B or latrunculin B, the microcell fraction is isolated by centrifugation and filtration. Microcells are fused with recipient cells using PEG or HVJ envelopes. Cells containing HACs are selected using the appropriate selective medium. (*B*) In retro-MMCT, donor cells are pre-transduced with lentiviruses encoding the MLV protein (blue circles on the surface of donor cells)

The main method used to transfer HACs and other vectors of 1 Mbp or more in length is microcell-mediated chromosome transfer (MMCT), which enables the transfer of these vectors from donor cells to target cells using the so-called microcells (Fig. 6A) [74]. In donor cells, the formation of micronuclei, which are individual chromosomes surrounded by a nuclear envelope, is initiated. For this purpose, donor cells are incubated with cytostatic agents, colcemid [75] or griseofulvin, and TN-16 [76, 77], which cause cell cycle arrest at the metaphase stage. A9 (mouse subcutaneous tissue) or CHO cells are used as donor cells [8]. Donor cells are then fragmented into microcells by treatment with actin filament assembly inhibitors (cytochalasin B [75] or latrunculin B [76, 77]), followed by prolonged centrifugation. The microcell fraction is isolated using filtration [75] or a percoll gradient [60]. Microcells are then fused with target cells using polyethylene glycol (PEG) [75] or the hemagglutinating virus of the Japan envelope (HVJ-E) [23, 56, 57]. A retro-MMCT method (Fig. 6B) based on the use of the murine leukemia virus (MLV) envelope protein demonstrated improved efficiency compared with that of the original method. The MLV protein on the surface of microcells mediates their binding to the plasma membrane protein present on the surface of almost all types of mammalian cells, thus increasing the efficiency of cell–microcell fusion [78]. Using this MMCT variant, the alphoid^tetO^-HAC was successfully transferred to human iPSCs [58]. It is important to note that various modifications of MMCT can be combined at its different stages, thereby increasing the efficiency of HAC transfer [12, 56, 58, 77, 78]. Cells bearing a target HAC are selected by culturing in the presence of antibiotics (blasticidin, G418, etc.) resistance to which is provided by the HAC.

**Fig. 7:**
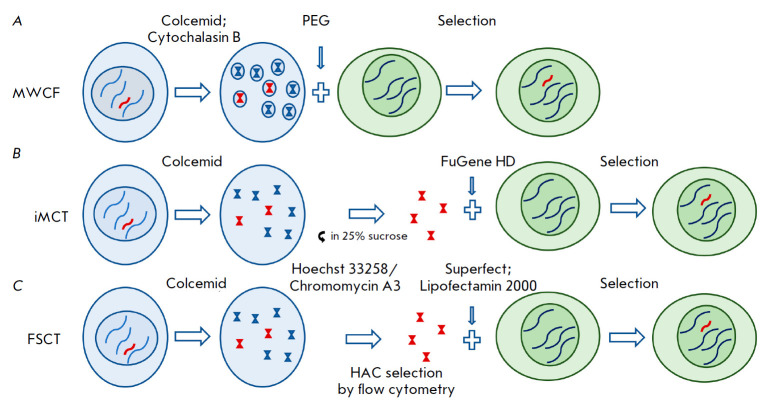
Methods of HAC transfer that do not use microcells. (*A*) MWCF – fusion of donor cells with recipient cells. (*B*) iMCT – transfection of isolated chromosomes into target cells using lipofection. Colcemid-pretreated donor cells are lysed, and HACs isolated on a sucrose gradient are transferred to recipient cells using the FuGene HD reagent. (*C*) In FSCT, HACs are stained with Hoechst 33258 and chromomycin A3, collected by flow cytometry, and transfected into target cells using lipofection

Apart from MMCT, there are also methods for HAC transfer that do not use microcells. For example, in micronucleated whole cell fusion (MWCF), donor cells are fused with target cells using PEG after successive exposure to colcemid and cytochalasin B [79]. This method was developed to transfer HACs from cells that are not resistant to long-term exposure to cytostatics. The advantage of this method is high (compared with MMCT) efficiency and ease of use. However, a major limitation of this method is the need to use for the fusion cells of different animal species. Isolated metaphase chromosome transfer (iMCT) allows HAC transfer from donor cells incapable of forming micronuclei. In this method, HACs are isolated from a lysate of colcemid-pretreated cells using separation in the sucrose gradient [80]. The isolated HACs are transfected into target cells using li pofection. This method has been sparsely used due to its low efficiency. Finally, Flow sorted chromosome transfer (FSCT) was developed for HACs containing C-G-rich sequences. In this case, HACs pretreated with Hoechst 33258 and chromomycin A3 dyes are isolated by flow cytometry (Fig. 7C). The isolated HACs are then transfected into target cells by lipofection [37, 81].

## CONCLUSION

Currently, HACs are considered promising expression vector systems. The unique properties of HACs are their inertness and autonomy in the genome of target cells and the ability to bear large-sized transgenes. These properties of HAC-based genetic vectors are in demand in many areas of modern biology and medicine. HACs have been used in the development of approaches to the reprogramming of cells into iPSCs, creation of transgenic animals, and the generation of experimental models for the treatment of genetic diseases. HACs have been also extensively used to study chromosome functions and chromosomal instability.

However, despite the huge demand for HACs, the technologies of their production and transfer still need significant improvement before their implementation and wide application in laboratory practice and biomedicine. First of all, the transfer of HACs to recipient cells remains laborious and inefficient. Successful optimization of the methods for HAC transfer to recipient cells will increase the overall value and use of these genetic vectors in research and therapeutic applications.

## Abbreviations

HAC: human artificial chromosome
BAC: bacterial artificial chromosome
YAC: yeast artificial chromosome
MMCT: microcell-mediated chromosome transfer
TAR: transformation-associated recombination
CENP-B: centromere protein B
ESCs: embryonic stem cells
iPSCs: induced pluripotent stem cells
CHO: Chinese hamster ovary
tetO: tetracycline operator
HSV-1: herpes simplex virus 1
HVJ-E: hemagglutinating virus of Japan envelope
MLV: murine leukemia retrovirus
TACF: telomere associated chromosome fragmentation
FSCT: flow sorted chromosome transfer
MWCF: micronucleated whole cell fusion
iMCT: isolated metaphase chromosome transfection
PEG: polyethylene glycol
HPRT: hypoxanthine-guanine phosphoribosyltransferase
FACS: fluorescence activated cell sorting
GFP: green fluorescent protein
DNA: deoxyribonucleic acid
bp: base pairs
